# Drinking hydrogen water and intermittent hydrogen gas exposure, but not lactulose or continuous hydrogen gas exposure, prevent 6-hydorxydopamine-induced Parkinson’s disease in rats

**DOI:** 10.1186/2045-9912-2-15

**Published:** 2012-05-20

**Authors:** Mikako Ito, Masaaki Hirayama, Kazuaki Yamai, Sae Goto, Masafumi Ito, Masatoshi Ichihara, Kinji Ohno

**Affiliations:** 1 Division of Neurogenetics, Center for Neurological Diseases and Cancer, Nagoya University Graduate School of Medicine, Nagoya, Japan; 2 Department of Pathophysiological Laboratory Sciences, Nagoya University Graduate School of Medicine, Nagoya, Japan; 3 Department of Biomedical Sciences, College of Life and Health Sciences, Chubu University, Kasugai, Japan; 4 Research Team for Mechanism of Aging, Tokyo Metropolitan Institute of Gerontology, Tokyo, Japan; 5 Division of Neurogenetics, Center for Neurological Diseases and Cancer, Nagoya University Graduate School of Medicine, 65 Tsurumai, Showa-ku, Nagoya, 466-8550, Japan

**Keywords:** Lactulose, Hydrogen water, Continuous hydrogen gas, Intermittent hydrogen gas, Parkinson’s disease, 6-Hydroxydopamine

## Abstract

**Background:**

Lactulose is a synthetic disaccharide that can be catalyzed only by intestinal bacteria in humans and rodents, and a large amount of hydrogen is produced by bacterial catalysis of lactulose. We previously reported marked effects of *ad libitum* administration of hydrogen water on prevention of a rat model of Parkinson’s disease (PD).

**Methods:**

End-alveolar breath hydrogen concentrations were measured in 28 healthy subjects and 37 PD patients, as well as in 9 rats after taking hydrogen water or lactulose. Six-hydroxydopamine (6-OHDA)-induced hemi-PD model was stereotactically generated in rats. We compared effects of hydrogen water and lactulose on prevention of PD. We also analyzed effects of continuous and intermittent administration of 2% hydrogen gas.

**Results:**

Hydrogen water increased breath hydrogen concentrations from 8.6 ± 2.1 to 32.6 ± 3.3 ppm (mean and SEM, *n* = 8) in 10 min in healthy subjects. Lactulose increased breath hydrogen concentrations in 86% of healthy subjects and 59% of PD patients. Compared to monophasic hydrogen increases in 71% of healthy subjects, 32% and 41% of PD patients showed biphasic and no increases, respectively. Lactulose also increased breath hydrogen levels monophasically in 9 rats. Lactulose, however, marginally ameliorated 6-OHDA-induced PD in rats. Continuous administration of 2% hydrogen gas similarly had marginal effects. On the other hand, intermittent administration of 2% hydrogen gas prevented PD in 4 of 6 rats.

**Conclusions:**

Lack of dose responses of hydrogen and the presence of favorable effects with hydrogen water and intermittent hydrogen gas suggest that signal modulating activities of hydrogen are likely to be instrumental in exerting a protective effect against PD.

## Background

Lactulose is a synthetic sugar that is made of fructose and galactose and is not absorbed by humans or rodents because of lack of an enzyme that catalyzes the disaccharide. Lactulose, however, can be digested by bacteria in the colon, and hydrogen is produced as a byproduct [1]. A hydrogen breath test after oral administration of lactulose is clinically applied to examine small intestinal bacterial overgrowth, which is an underlying mechanism of irritable bowel syndrome [2]. Lactulose is also used to treat chronic constipation [3] and hepatic encephalopathy [4]. Lactulose is able to ameliorate dextran sulfate sodium (DSS)-induced intestinal inflammation in rats [5]. The effect is likely due to alteration of intestinal microflora, because lactulose increases intestinal levels of *Lactobacillus*[6] and *Lactobacillus* prevents development of colitis in interleukin 10-deficient mice [7]. The effect of lactulose on DSS-induced colitis can also be ascribed to hydrogen production in the colon, because markers of oxidative stress are reduced in the lactulose-administered rats [5]. In addition, Chen and colleagues recently hypothesized that lactulose potentially ameliorates cerebral infarction by producing intestinal hydrogen [8].

We previously reported that *ad libitum* administration of hydrogen water abolishes development of parkinsonian symptoms in a rat model of 6-hydroxydopamine (6-OHDA)-induced Parkinson’s disease (PD) [9]. As drinking a large amount of water is not easily accommodated by PD patients, we examined whether lactulose is able to increase breath hydrogen levels in PD patients. We additionally tested effects of lactulose on breath hydrogen levels and on development of 6-OHDA-induced PD in rats. Lactulose efficiently increased breath hydrogen levels in healthy subjects, PD patients, and rats. Lactulose, however, marginally ameliorated development of PD in rats. We also demonstrated that continuous inhalation of hydrogen gas had marginal effects, whereas intermittent inhalation had variable but overt effects on prevention of PD in rats.

## Materials and methods

### Hydrogen preparations

We made hydrogen-saturated water (1.6 ppm or 0.8 mM) for humans using the AquelaBlue electrolysis instrument (Miz Co. Ltd., Fujisawa, Japan). Hydrogen water for rats was provided by Blue Mercury Inc. (Tokyo, Japan). Pure air (200 ml, Japan Fine Products, Kawasaki, Japan) was equilibrated with 1 ml of hydrogen water, and the hydrogen concentration in the air was measured by a gas chromatograph connected to a semiconductor gas detector (EAGanalyzer GS-23, SensorTec Co. Ltd., Ritto, Shiga, Japan). The hydrogen concentrations of the AquelaBlue water were 1.4–1.6 ppm and those of the Blue Mercury water were 1.0–1.2 ppm. We purchased lactulose from Kowa Pharmaceuticals (Nagoya, Japan).

### Human studies

The human studies were approved by the Ethical Review Committee of the Nagoya University Graduate School of Medicine. Twenty-eight healthy subjects (38 ± 10 years; mean and SD) and 37 PD patients (59 ± 9 years) participated in the studies after appropriate informed consent was obtained. The participants refrained from all food, supplements, and drugs, except water, for at least 12 hours before the studies. For studies of hydrogen water, the healthy participants rested in a sitting position for at least 30 min and took 200 ml of hydrogen-saturated water. End-alveolar breath was obtained in a closed aluminum bag every 5 min for 60 min. For studies of lactulose, the healthy participants and PD patients took 6 g lactulose in 50 ml of water, which was the conventional dose in clinical practice. End-alveolar breath was obtained in a closed aluminum bag every 10 min for 180 min. The breath was immediately transferred to a gas-tight glass syringe and 1 ml was injected into EAGanalyzer GS-23 to measure hydrogen concentrations.

### Measurement of end-alveolar hydrogen concentrations in rats

All rat experiments were approved by the Animal Care and Use Committee of the Nagoya University Graduate School of Medicine. Male Sprague–Dawley rats (~300 g) were anesthetized by an intraperitoneal injection of 330 mg/kg of chloral hydrate, and were inserted with a tracheal tube following tracheotomy. Lactulose (1.3 g/kg) was then intragastrically administrated through a gastric tube. We aspirated 1 ml tracheal gas and immediately injected 2 ml pure air to the trachea. The 2 ml pure water was aspirated in two seconds and was transferred to a closed aluminum bag containing 20 ml of pure air. The hydrogen concentrations were measured with EAGanalyzer GS-23.

### A rat model of 6-OHDA-induced Parkinson’s disease

We stereotactically infused 20 μg of 6-OHDA in 2 μl into the right striatum of seven-week-old male Sprague–Dawley rats (~250 g) as previously described [9]. At four weeks after the surgery, we counted the number of clockwise turns in 30 min after intraperitoneal administration of 5.0 mg/kg of methamphetamine (Dainippon Sumitomo Pharmaceuticals, Osaka, Japan) The numbers of tyrosine hydroxylase (TH)-positive cells at the substantia nigra were counted by two blinded investigators at four weeks [9].

We used five different protocols of hydrogen administration. For controls (*n* = 5) and hydrogen water (*n* = 5), we used the data that we reported previously [9]. To confirm that we could still observe prominent effects of hydrogen water, we analyzed two additional rats with control water and two with hydrogen water. Lactulose (3.0 g) was dissolved in 100 ml drinking water. As ~250-g rats took ~25 ml of water per day, the rats took ~3.0 g/kg/day of lactulose, which was 10 times higher than the conventional dose of 0.3 g/kg/day for humans (18 g/day for 60 kg body weight). As the safe maximum dose of lactulose for rats was 12 g/kg/day, the rats well tolerated ~3.0 g/kg/day of lactulose without overt adverse effects including diarrhea. For continuous administration of 2% hydrogen gas, two rats were placed in a 20-liter air-tight chamber, which was continuously supplied with 8 liter/min of 2% hydrogen gas (Iwatani, Tokyo, Japan). For intermittent administration of 2% hydrogen gas, the 20-liter air-tight chamber was supplied serially with 2% hydrogen gas for 15 min and then room air for 45 min at 8 liter/min using a time controller. The one-hour cycle was repeated 12 times from 6 pm to 6 am to recapitulate the habit of drinking water once every hour in the dark [10]. Each hydrogen administration protocol was started 1 week before the surgery. We used the Prizm 4.0c (GraphPad Software, La Jolla, CA) for statistical analyses.

## Results

### Lactulose increased breath hydrogen in humans

**Figure 1:**
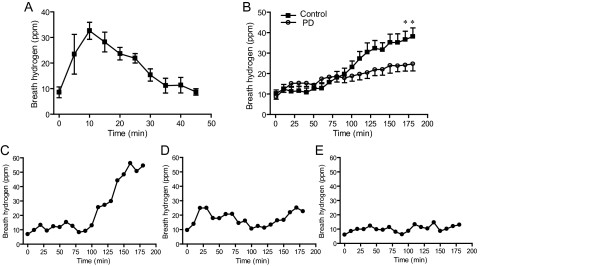
**Temporal profiles of end-alveolar breath hydrogen concentrations in humans. (A)** Breath hydrogen concentrations after drinking 200 ml hydrogen-saturated water in 8 healthy subjects. Mean and SEM are indicated. **(B)** Average breath hydrogen concentrations after taking 6 g lactulose in 28 healthy subjects and 37 PD patients. The two curves are statistically different (*P* < 0.0001 by two-way ANOVA). *P < 0.05 by Bonferroni post-hoc test. Mean and SEM are indicated. Three representative temporal patterns of monophasic **(C)**, biphasic **(D)**, and no increases **(E)** of breath hydrogen concentrations after taking 6 g lactulose in PD patients.

We first examined end-alveolar breath hydrogen concentrations after taking 200 ml hydrogen-saturated water in eight healthy subjects (Figure 1A). The hydrogen concentrations increased from 8.6 ± 2.1 (mean and SEM, *n* = 8) ppm to 32.6 ± 3.3 ppm in 10 min. The breath hydrogen concentration, however, became the basal levels in 45 min.

We next examined lactulose-induced hydrogen production in 28 healthy subjects. After taking 6 g lactulose in 50 ml water, end-alveolar breath hydrogen concentrations started to increase at 70 min and reached 38.0 ± 4.2 (mean and SEM, *n* = 28) ppm at 180 min (Figure 1B). The hydrogen concentrations did not reach a plateau in the observation period of 180 min. PD patients (*n* = 37) also showed similar increases of breath hydrogen concentrations, but the average increases were about half of the healthy subjects (*P* < 0.0001 by two-way ANOVA) (Figure 1B). Inspection of temporal profiles of hydrogen concentrations in healthy subjects and PD patients revealed that there are three temporal patterns (Figure 1C, D, and E). First, in 20 controls (71%) and 10 PD patients (27%), the hydrogen concentrations gradually increased in a monophasic manner (Figure 1C). Second, in 4 controls (14%) and 12 PD patients (32%), the hydrogen concentrations increased in a biphasic manner (Figure 1D). Third, in 4 controls (14%) and 15 PD patients (41%), the hydrogen concentrations remained essentially unchanged after lactulose intake (Figure 1E). The Fisher’s exact test revealed that the temporal patterns are different between healthy subjects and PD patients (*P* < 0.005).

### Lactulose increased breath hydrogen in rats

**Figure 2:**
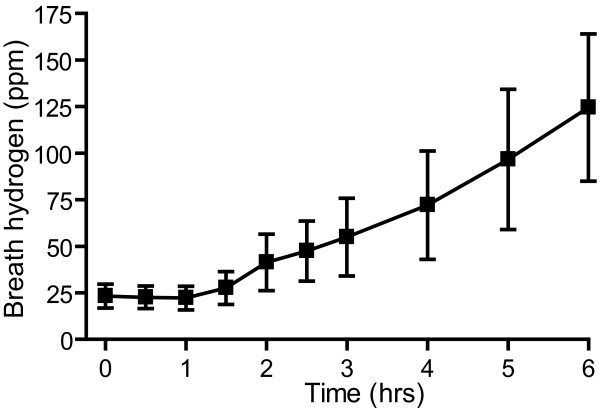
**Temporal profiles of end-alveolar breath hydrogen concentrations after taking lactulose in rats.** Average breath hydrogen concentrations after intragastric administration of 1.3 g/kg lactulose in rats. Mean and SEM of 9 rats are indicated.

We next hoped to examine effects of lactulose-induced hydrogen production on prevention of PD in rats. We first examined whether lactulose was able to increase breath hydrogen concentrations in rats. We intragastrically administered 1.3 g/kg lactulose to ~300 g rats and measured breath hydrogen concentrations (Figure 2). The basal hydrogen concentrations were 23.3 ± 6.4 ppm (mean and SEM, *n* = 9). The concentrations started to increase at 1.5 hrs after ingestion of lactulose, and reached 124.5 ± 39.5 ppm at 6 hrs (Figure 2).

### Lactulose marginally ameliorates PD in rats

**Figure 3:**
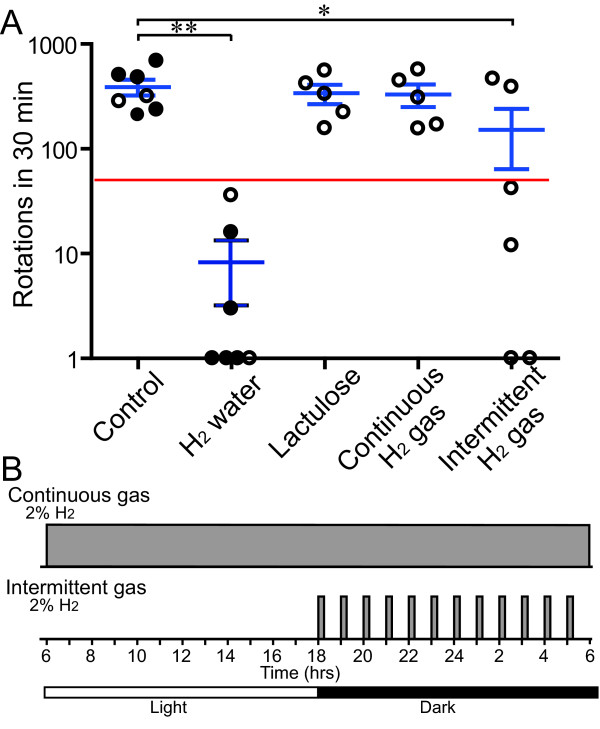
**Behavioral assays of 6-OHDA-infused rat model of hemi-Parkinson’s disease.** Circles represent the number of turns in 30 min after intraperitoneal injection of methamphetamine in each rat in each group. Mean and SEM are indicated in blue. A red line represents a putative threshold of 50 turns to conclude that development of PD is ameliorated. Results of five rats with control water and five with hydrogen water that we reported previously are indicated by closed circles [9]. Two additional rats with control water and two with hydrogen water are newly analyzed for this report (open circles). The five groups are statistically different (*P* < 0.001) by one-way ANOVA. The Dunnett’s multiple comparison post-hoc test reveals that hydrogen water (**P < 0.01) and intermittent hydrogen gas (*P < 0.05) are different from controls.

We examined effects of lactulose on prevention of PD in rats. We infused 6-OHDA in the right striatum of 7-week-old male SD rats to make hemi-PD. Intraperitoneal injection of methamphetamine facilitates release of synaptic dopamine at the dopaminergic nerve terminals at the striatum, which causes the rat to turn clockwise. We counted the number of clockwise turns in 30 min at 4 weeks after the surgery, and quantified effects of hydrogen on prevention of development of PD (Figure 3A). We previously reported that control water failed to prevent PD development and the rats rotated 230 or more times in 30 min, whereas hydrogen water efficiently prevented development of PD and the rats rotated less than 50 times in 30 min [9]. We examined two additional rats with control water and two with hydrogen water to confirm that hydrogen water still had marked effects in our experimental system (Figure 3A).

The PD model rats (*n* = 5) started to take ~3.0 g/kg/day lactulose in drinking water one week before the surgery. As 60-kg humans take a maximum of 36 g/day of lactulose for hepatic encephalopathy, 3.0 g/kg/day is 5 times more than the amount that is used for humans. The rats, however, showed no side effects. We confirmed that the breath hydrogen concentrations at 4 weeks were as high as 120.0 ± 12.6 ppm (mean and SEM, *n* = 3) in the PD model rats. We counted the number of methamphetamine-induced clockwise turns, and found that lactulose marginally ameliorated motor deficits but without statistical significance (Figure 3A). We previously reported that infusion of 6-OHDA reduced the number of TH-positive cells at the substantia nigra to 40.2 ± 10.6% (mean and SD, *n* = 5), whereas hydrogen water increased the ratio to 83.0 ± 10.2% [9]. Lactulose failed to rescue the death of dopaminergic cells at the substantia nigra and the number of TH-positive cells remained as low as 37.0 ± 6.5% (mean and SD, *n* = 5), which was not statistically different from that of control water.

### Effects of continuous and intermittent hydrogen gas on a rat model of PD

In order to simulate lactulose administration, the PD model rats (*n* = 5) were placed in a 2%-hydrogen chamber one week before the surgery and stayed for four weeks after the surgery. As we observed with lactulose, the continuous administration of hydrogen gas marginally ameliorated the number of clockwise turns but without statistical significance (Figure 2A).

The differential effects of hydrogen water and continuous administration of hydrogen by lactulose and hydrogen gas prompted us to hypothesize that intermittent exposure to hydrogen may be essential to exert beneficial effects for PD. We thus developed a time-controlled gas chamber system, in which rats were serially exposed to 2% hydrogen for 15 min and then room air for the remaining 45 min (Figure 2B). The one-hour cycle was repeated from 6 pm to 6 am to recapitulate the rats’ habit of drinking water in the dark. Rats (*n* = 6) were placed in the intermittent hydrogen gas chamber one week before the surgery and stayed for four weeks after the surgery. Four of the six rats favorably responded to intermittent hydrogen administration and the numbers of clockwise turns were decreased to less than 50, whereas the remaining two rats showed no effects (Figure 2A). The mean and SD of the numbers of turns were 151.8 ± 216.3, which was significantly lower than those of controls (*P* < 0.05).

## Discussion

Lactulose efficiently increased the breath hydrogen levels in healthy subjects. Although the temporal profiles of breath hydrogen concentrations were more variable in PD patients, the increased hydrogen concentrations was on average about half of that of healthy subjects. We observed biphasic increases of breath hydrogen in 12 of the 37 PD patients. Biphasic increases of breath hydrogen in a hydrogen breath test suggests small intestinal bacterial overgrowth (SIBO), but the increases of hydrogen concentrations in the PD patients were less than a commonly used threshold of 20 ppm for SIBO [2]. The difference in temporal profiles of the breath hydrogen concentrations between controls and PD patients, however, might be due to the difference in the ages of the two groups (38 ± 10 years vs. 59 ± 9 years). We additionally confirmed that rats also had increased breath hydrogen levels in a monophasic manner after taking lactulose, which underscored a notion that intestinal bacteria digest lactulose and produce hydrogen in humans and rats.

In contrast to marked effects of *ad libitum* administration of hydrogen water on a rat model of PD, lactulose and continuous administration of 2% hydrogen gas had marginal effects. On the other hand, intermittent administration of 2% hydrogen gas had variable but overt effects. When a 60-kg person drinks 1000 ml of 72% saturated hydrogen water, the hydrogen concentration of the body water is expected to become 0.8 mmoles x 72% / (60 kg x 60%) = 0.016 mM (2% saturation), which is identical to the hydrogen concentration achieved by a person staying in a 2% hydrogen chamber. Although hydrogen in drinking water can bind to glycogen in the liver [11], hydrogen easily dissipates in exhalation (Figure 1A). On the other hand, a person in a 2% hydrogen chamber is exposed to 2% hydrogen for 24 hrs. The area under the curve of hydrogen concentrations in a hydrogen chamber is estimated to be thus ~100 times more than that with hydrogen water. Although a ~250-g SD rat takes ~25 ml of water per day, which is six times more than the amount that humans take, the rats placed in a hydrogen chamber were still exposed to much more amounts of hydrogen compared to the rats that took hydrogen water. Nevertheless, we observed a prominent protective effect against PD with hydrogen water but not with continuous inhalation of 2% hydrogen gas or lactulose. Lack of the dose response is also supported by our observation that intermittent inhalation of 2% hydrogen gas was more protective than continuous inhalation, although the amount of hydrogen was eight times less with intermittent hydrogen administration.

Another line of evidence that supports lack of the dose response of hydrogen is intestinal production of hydrogen. Although no mammalian cells can produce hydrogen endogenously, hydrogen is produced by intestinal bacteria carrying hydrogenase. Production of hydrogen by lactulose is also achieved by these bacteria. We humans are able to make a maximum of 12 liters of hydrogen in our intestines [12,13]. Nevertheless, oral administration of a small amount of hydrogen exhibits prominent effects. In a mouse model of Concanavalin A-induced hepatitis, elimination of intestinal bacteria by a cocktail of antibiotics worsened the hepatitis [14]. Restitution of a hydrogenase-negative strain of *E. coli* had no effects, whereas that of a hydrogenase-positive stain of *E. coli* ameliorated the hepatitis. This is the only report that addressed a beneficial effect of intestinal bacteria, but they also demonstrated that drinking hydrogen water was more effective than restitution of hydrogenase-positive bacteria. In addition to lactulose that we tested in our studies, we can easily increase hydrogen concentrations in our bodies by an α-glucosidase inhibitor, acarbose [15]; milk for lactase-deficient adults that constitute 75% of the worldwide population [16]; and an ingredient of curry, turmeric, which enhances the bowel movement [17]. These compounds, however, are unlikely to be effective for PD.

Ohsawa and colleagues reported prominent effects of inhaled hydrogen gas for a rat model of cerebral infarction [18]. They demonstrated that the prominent effect of hydrogen is ascribed to a specific scavenging activity of hydroxyl radicals. Effects of inhaled hydrogen gas have been reported in 21 additional diseases, and similarly effects of drinking hydrogen water have been reported in 18 diseases since then [19]. Direct comparison of effects of *ad libitum* administration of hydrogen-saturated water and inhalation of 1% hydrogen gas on a mouse model of cisplatin-induced nephropathy demonstrated that the two modalities of hydrogen administration had similar effects [20]. A small amount of hydrogen is likely to be sufficient to ameliorate nephropathy and PD in rats, but an excessive amount of hydrogen may somehow negate a favorable effect for PD, although no adverse effects of hydrogen have been reported to date. Further studies are required to prove whether the lack of dose response is disease-specific or not.

We previously reported that hydrogen attenuates phosphorylation of FcϵRI-associated Lyn and its downstream signaling molecules in rat RBL-2H3 mast cells [21]. We also demonstrated that hydrogen ameliorates an immediate-type allergic reaction not by a radical-scavenging activity but by direct modulation of signaling pathway(s). In addition, using murine RAW264 macrophage cells, we demonstrated that hydrogen reduces lipopolysaccharide/interferon-γ-induced nitric oxide (NO) production [22]. We found that hydrogen inhibits phosphorylation of ASK1 and its downstream signaling molecules, p38 MAP kinase, JNK, and IκBα without affecting ROS production by NADPH oxidase. Both studies point to a notion that hydrogen is a gaseous signaling modulator. The initial increase of hydrogen concentrations in rats drinking hydrogen water was expected to be faster than that of intermittent administration of 2% hydrogen gas, because we added 8 liter per min of 2% hydrogen gas to a 20-liter chamber. Prominent effects that we observed with hydrogen water may be ascribed to the pulsatile increase of hydrogen concentrations in rats.

## Conclusions

Lactulose is a safe and tolerable source of hydrogen production for healthy subjects and PD patients. Although lactulose also increases hydrogen levels in rats, lactulose and continuous inhalation of 2% hydrogen gas have marginal effects on prevention of 6-OHDA-induced PD. On the other hand, intermittent inhalation of hydrogen gas variably but overtly prevents development of PD, but not as efficiently as *ad libitum* administration of hydrogen water.

## Abbreviations

PD: Parkinson’s disease; 6-OHDA: 6-hydroxydopamine.

## Competing interests

We have no competing interest to disclose.

## Authors’ contributions

MI^1^ performed rat experiments. MH and SG examined patients and acquired data. KY analyzed breath hydrogen in rats. MI^1^, MH, and KO organized data and wrote the paper. MH, MI^3^, MI^4^, and KO conceived the study. All authors read and approved the final manuscript.

## References

[B1] Florent C Flourie B Leblond A Rautureau M Bernier JJ Rambaud JC Influence of chronic lactulose ingestion on the colonic metabolism of lactulose in man (an in vivo study) J Clin Invest 1985 75 608 613 10.1172/JCI111738 3973020 PMC423537

[B2] Ford AC Spiegel BM Talley NJ Moayyedi P Small intestinal bacterial overgrowth in irritable bowel syndrome: systematic review and meta-analysis Clin Gastroenterol Hepatol 2009 7 1279 1286 10.1016/j.cgh.2009.06.031 19602448

[B3] Voskuijl W de Lorijn F Verwijs W Hogeman P Heijmans J Makel W Taminiau J Benninga M PEG 3350 (Transipeg) versus lactulose in the treatment of childhood functional constipation: a double blind, randomised, controlled, multicentre trial Gut 2004 53 1590 1594 10.1136/gut.2004.043620 15479678 PMC1774276

[B4] Patil DH Westaby D Mahida YR Palmer KR Rees R Clark ML Dawson AM Silk DB Comparative modes of action of lactitol and lactulose in the treatment of hepatic encephalopathy Gut 1987 28 255 259 10.1136/gut.28.3.255 3570029 PMC1432706

[B5] Rumi G Tsubouchi R Okayama M Kato S Mozsik G Takeuchi K Protective effect of lactulose on dextran sulfate sodium-induced colonic inflammation in rats Dig Dis Sci 2004 49 1466 1472 15481321 10.1023/b:ddas.0000042248.48819.ad

[B6] Hoffmann K Mossel DA Korus W Van De Kamer JH Studies on the Mechanism of Action of Lactulose in the Intestine Klin Wochenschr 1964 42 126 130 10.1007/BF01479054 14152610

[B7] Madsen KL Doyle JS Jewell LD Tavernini MM Fedorak RN Lactobacillus species prevents colitis in interleukin 10 gene-deficient mice Gastroenterology 1999 116 1107 1114 10.1016/S0016-5085(99)70013-2 10220502

[B8] Chen X Zhai X Kang Z Sun X Lactulose: an effective preventive and therapeutic option for ischemic stroke by production of hydrogen Med Gas Res 2012 2 3 10.1186/2045-9912-2-3 22309834 PMC3298790

[B9] Fu Y Ito M Fujita Y Ito M Ichihara M Masuda A Suzuki Y Maesawa S Kajita Y Hirayama M Molecular hydrogen is protective against 6-hydroxydopamine-induced nigrostriatal degeneration in a rat model of Parkinson’s disease Neurosci Lett 2009 453 81 85 10.1016/j.neulet.2009.02.016 19356598

[B10] Johnson RF Johnson AK Light-dark cycle modulates drinking to homeostatic challenges Am J Physiol 1990 259 R1035 R1042 2240263 10.1152/ajpregu.1990.259.5.R1035

[B11] Kamimura N Nishimaki K Ohsawa I Ohta S Molecular Hydrogen Improves Obesity and Diabetes by Inducing Hepatic FGF21 and Stimulating Energy Metabolism in db/db Mice Obesity (Silver Spring) 2011 19 1396 1403 10.1038/oby.2011.6 21293445

[B12] Christl SU Murgatroyd PR Gibson GR Cummings JH Production, metabolism, and excretion of hydrogen in the large intestine Gastroenterology 1992 102 1269 1277 1551534

[B13] Strocchi A Levitt MD Maintaining intestinal H2 balance: credit the colonic bacteria Gastroenterology 1992 102 1424 1426 1551553 10.1016/0016-5085(92)90790-6

[B14] Kajiya M Sato K Silva MJ Ouhara K Do PM Shanmugam KT Kawai T Hydrogen from intestinal bacteria is protective for Concanavalin A-induced hepatitis Biochem Biophys Res Commun 2009 386 316 321 10.1016/j.bbrc.2009.06.024 19523450

[B15] Suzuki Y Sano M Hayashida K Ohsawa I Ohta S Fukuda K Are the effects of alpha-glucosidase inhibitors on cardiovascular events related to elevated levels of hydrogen gas in the gastrointestinal tract? FEBS Lett 2009 583 2157 2159 10.1016/j.febslet.2009.05.052 19505462

[B16] Shimouchi A Nose K Yamaguchi M Ishiguro H Kondo T Breath Hydrogen Produced by Ingestion of Commercial Hydrogen Water and Milk Biomarker Insights 2009 4 27 32 19652760 10.4137/bmi.s2209 PMC2716677

[B17] Shimouchi A Nose K Takaoka M Hayashi H Kondo T Effect of Dietary Turmeric on Breath Hydrogen Dig Dis Sci 2009 54 1725 1729 10.1007/s10620-008-0550-1 19034660

[B18] Ohsawa I Ishikawa M Takahashi K Watanabe M Nishimaki K Yamagata K Katsura K Katayama Y Asoh S Ohta S Hydrogen acts as a therapeutic antioxidant by selectively reducing cytotoxic oxygen radicals Nat Med 2007 13 688 694 10.1038/nm1577 17486089

[B19] Ohta S Nakao A Ohno K The 2011 Medical Molecular Hydrogen Symposium: An inaugural symposium of the journal Medical Gas Research Med Gas Res 2011 1 10 10.1186/2045-9912-1-10 22146082 PMC3231937

[B20] Nakashima-Kamimura N Mori T Ohsawa I Asoh S Ohta S Molecular hydrogen alleviates nephrotoxicity induced by an anti-cancer drug cisplatin without compromising anti-tumor activity in mice Cancer Chemother Pharmacol 2009 64 753 761 10.1007/s00280-008-0924-2 19148645

[B21] Itoh T Fujita Y Ito M Masuda A Ohno K Ichihara M Kojima T Nozawa Y Ito M Molecular hydrogen suppresses FcepsilonRI-mediated signal transduction and prevents degranulation of mast cells Biochem Biophys Res Commun 2009 389 651 656 10.1016/j.bbrc.2009.09.047 19766097

[B22] Itoh T Hamada N Terazawa R Ito M Ohno K Ichihara M Nozawa Y Molecular hydrogen inhibits lipopolysaccharide/interferon gamma-induced nitric oxide production through modulation of signal transduction in macrophages Biochem Biophys Res Commun 2011 411 143 149 10.1016/j.bbrc.2011.06.116 21723254

